# Bacterial exotoxins in medicine: potential value and perspectives

**DOI:** 10.7150/ijms.110104

**Published:** 2025-03-31

**Authors:** Yuming Xiao, Zhou Yan, Fangyuan Ren, Yurong Tan

**Affiliations:** 1 College of Life Sciences, Hunan Normal University, Changsha 410081, China.; 2 Department of Obstetrics, Zhuzhou Hospital Affiliated to Xiangya School of Medicine, Central South University, Zhuzhou 410017, China.; 3 Department of Medical Microbiology, Central South University Changsha 410083, Hunan, China.

**Keywords:** bacterial exotoxins, disease diagnosis, immunotherapy, vaccine development, immunotoxins

## Abstract

Bacterial exotoxins are protein- or peptide-based substances secreted by bacteria with high toxicity and specificity. They have diverse mechanisms of action on host cells, leading to host injury by destroying the cellular structure or interfering with physiological functions. With the continuous progress in biotechnology, bacterial exotoxins have broad application prospects in immunotherapy, vaccine development, drug design, and other fields. Appropriate modification of exotoxins can lead to the preparation of highly efficient immune agents and targeted drugs, which brings new hope for overcoming difficult diseases. This study provides a comprehensive and in-depth introduction to the application of bacterial exotoxins in the field of medicine and further explores the potential value of bacterial exotoxins, which is expected to make greater contributions to human health in the future.

## Introduction

Bacteria can cause diseases through two mechanisms: invasive inflammation and toxin production. Bacterial toxins are an important cause of diseases and are responsible for most symptoms and lesions during infection. These toxins can be divided into two groups: exotoxins, which are protein- or peptide-based toxic substances secreted by bacteria, and endotoxins, which are usually found within the cell wall and released into host tissues after cell death. Most Gram-positive and some Gram-negative bacteria are capable of producing exotoxins, which are proteins of chemical nature that give them unique physical and chemical properties, such as heat resistance, poor stability, and strong antigenicity. These properties not only determine the mode of action of exotoxins in the bacterial pathogenic process but also provide a theoretical basis for their application in many fields, such as medicine and biology. From a medical perspective, bacterial exotoxins are closely linked to the development of many diseases. For example, the Tetanospasmin produced by *Tetanus bacillus* can specifically block the release of inhibitory neurotransmitters, resulting in mandatory spasm of skeletal muscles; the botulinum toxin secreted by *Clostridium botulinum* can inhibit the release of acetylcholine, triggering muscle relaxation paralysis, which is a serious threat to the life and health of patients.

Bacterial exotoxins have high toxicity and specificity, and have various mechanisms of action on host cells, through the destruction of cell structure or interference with physiological functions that cause host damage. The action of exotoxins can occur far from the site of infection and can diffuse into the host. Although the mechanism of action of bacterial exotoxins remains complex, they share a common mechanism of action. Exotoxins are classified into three types according to their structure and function: i) superantigens, ii) membrane-disrupting toxins, and iii) A-B toxins.

### Type 1 toxins (superantigens)

Type 1 toxins act outside of the cell. Superantigens (SAgs) belong to this class of toxins. Antigen-presenting cells (APCs), such as macrophages, break down antigens into peptides that bind to MHC II molecules to form the "MHC-peptide complex," which is recognized by a small number of TH cells with specific receptors. However, superantigens bind non-specifically to MHC II molecules and T-cell receptors (TCRs) on the surface of APCs, non-specifically activating a large number of T-cells and releasing a large number of cytokines, including interleukin-2 (IL-2). Significantly elevated levels of IL-2 in the blood can trigger a range of symptoms such as nausea, vomiting, malaise, diarrhea, fever, and shock and may even lead to death 1. For example, Toxic Shock Syndrome Toxin-1 (TSST-1), produced by Staphylococcus aureus, is associated with toxic shock syndrome. It binds to T-cell receptors and MHC class II molecules, activating a large number of T cells and triggering a strong immune response, which can lead to severe symptoms such as fever and shock.

### Type II toxins (membrane-damaging toxins)

Type 2 toxins act directly on the cell membrane without transferring to the interior of the target cell, targeting eukaryotic cell membranes (e.g., phospholipases, lecithinases, and pore-forming cytotoxins). These toxins disrupt the integrity of the mammalian cell membranes, leading to cell lysis. There are two types of membrane-disrupting toxins: one acts by forming protein channels in the plasma membrane. Because the osmotic pressure of the host cell's cytoplasm is higher than that of the surrounding environment, the formation of channels leads to the creation of holes in the cell membrane, triggering cellular swelling. This eventually leads to cell lysis because the cell membrane is unable to withstand the sudden influx of large amounts of fluid. For example, alpha-hemolysin (from *Staphylococcus aureus*) forms hexameric or heptameric pores in the cell membrane, causing an abnormal flow of ions (e.g., calcium, potassium), lysing erythrocytes, and destroying host cells 2. The second type is enzymatic toxins, such as Phospholipase C, which disrupt the integrity of membrane phospholipids. The charged groups at the head of the lipid bilayer stabilize the host cell membrane, and by removing these head groups, phospholipases destabilize the membrane structure, triggering host cell lysis 3. Other phospholipases also play a role in destabilizing the host cell membrane by cleaving phospholipid molecules at different locations.

### Type III toxins (A-B toxins)

**Figure 1:**
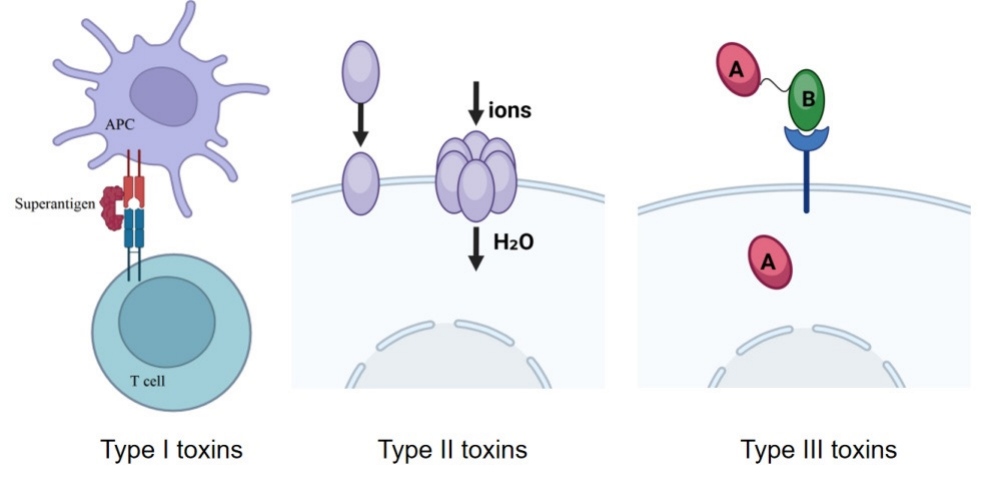
Classification of bacterial exotoxins.

Type 3 toxins act on intracellular targets and are usually composed of A-B structures: the A subunit (active subunit) is the enzymatic component that activates or inactivates intracellular targets or signaling pathways; the B subunit (binding subunit) is the binding component that is responsible for toxin binding and internalization to target cells, binding the exotoxin to the specific receptor on the cell membrane, for example, Diphtheria Toxin: the Binding subunit is the binding component responsible for binding and internalization of toxin to target cells. The A subunit blocks protein synthesis through ADP-ribosylation of the host elongation factor 2 (EF-2) 4 (Fig. 1).

An in-depth investigation of the pathogenic mechanisms of bacterial exotoxins will help us understand the pathological processes of these diseases more precisely, thus providing a basis for the development of targeted therapeutic approaches. In biological research, bacterial exotoxins are powerful tools for studying cellular processes. Owing to their high affinity and selective effects on specific cellular targets, they are capable of interfering with normal cellular physiological functions, such as signal transduction, protein synthesis, and nucleic acid metabolism. By analyzing the mechanism of action of exotoxins, we can gain insights into the complex molecular networks and signaling pathways within the cell, providing important clues to reveal the basic laws of life activities.

## Application of bacterial exotoxins in medical field

### Application in disease diagnosis

Bacterial exotoxins can be used as key markers in disease diagnosis. Immunoassays are commonly used methods, such as enzyme-linked immunosorbent assay (ELISA), the principle of which is based on antigen-antibody specific binding reactions. The known exotoxin antibody is immobilized on the surface of the solid-phase carrier and added to the sample to be tested; if the corresponding exotoxin exists in the sample, the two will specifically bind to form an antigen-antibody complex, then the enzyme-labeled secondary antibody is added, the enzyme catalyzes the color development of the substrate, and the exotoxin content can be determined qualitatively or quantitatively based on the color depth to assist in the diagnosis of the disease 5. In the diagnosis of Shiga toxin-producing Escherichia coli (STEC), the ELISA method for detecting the toxin has extremely high sensitivity and specificity in aiding the diagnosis of E. coli infections. Compared with the traditional diagnosis of clinical symptoms, exotoxin-based diagnostic technology has higher accuracy and specificity, especially for the diagnosis of patients with atypical symptoms or latent period, and the detection speed is faster, which can provide time for timely treatment and reduce the mortality and disability rate of patients. However, this method also has certain limitations, such as the possibility of interference from the patient's autoimmune status, recent vaccination history, and other factors, resulting in false-positive or false-negative results.

In addition, immunofluorescence technology uses fluorescein-labeled exotoxin antibody, which binds to the exotoxin in the sample. Specific fluorescent signals can be observed under a fluorescence microscope, realizing the localization and detection of bacterial exotoxins, which can help to quickly determine the infection, such as in the early diagnosis of certain bacterial infectious diseases, and provide a basis for prompt treatment. In the diagnosis of botulism, the detection of botulinum toxin in patient serum through a neutralization test can quickly confirm botulism.

### Immunotherapeutic applications of exotoxins

Studying the molecular mechanisms by which toxins interact with the immune system will contribute to the development of effective therapeutic and immunological intervention strategies. One of the most effective ways to prevent human and animal diseases is the widespread use of vaccines. Many human pathogens produce exotoxins, and vaccines against exotoxins have been shown to be of great value to public health. For example, research on vaccines against the exotoxin of *Pseudomonas aeruginosa* will address the problem of drug resistance caused by this bacterium 6. Exotoxin vaccines are categorized into the following types based on their preparation techniques and application forms:

#### Toxoid vaccines

Using the immunogenicity of exotoxins, the formation of toxoids by chemical inactivation of exotoxins stimulates the production of neutralizing antibodies in the host, thus protecting the host from toxin-associated diseases, such as *Staphylococcus aureus* leukocidin and hemolysin, which play an important role in the pathogenesis of staphylococci by killing the host's immune cells and erythrocytes. Gamma-hemolysin A [HlgA], leukocidin S [LukS], leukocidin AE323AB [LukAE323AB], and alpha-hemolysin H35L [HlaH35L] are the optimal vaccine components for the protection of human erythrocytes and neutrophils against staphylococcal PFTs 7. Toxoid vaccines have been widely used since the 1920s and are known to trigger a strong and protective humoral immune response after initial vaccination but require multiple booster injections to establish long-term immunity 8. Unlike vaccines against whole pathogens or viral particles that contain multiple immunogenic epitopes, toxoid vaccines contain only a single protein. However, multiple antigenic epitopes are also present in a single protein, some of which are more immunogenic than the others.

Toxoid vaccines can also stimulate T cell responses, which helps enhance protection against disease 9. When a toxoid vaccine is administered, APCs such as dendritic cells normally process the toxoid and present peptide antigens on their cell surface via major histocompatibility complex class II molecules (MHC-II). These MHC-II-peptide complexes act as signals to T cells, especially CD4^+^ T cells, and trigger a series of immune responses that activate B cells and trigger cytokine secretion in the host, which in turn leads to the differentiation of B cells into antibody-producing plasma and memory B cells 10.

A classic example of a toxoid vaccine is diphtheria toxoid, which consists of a chemically inactivated toxoid combined with an adjuvant such as aluminum hydroxide (alum) 11 and is effective in preventing diphtheria. The toxoid in the vaccine is taken up and processed by antigen-presenting cells (APCs). Helper T cells recognize the toxoid fragments and become activated. The activated T cells then stimulate B cells to produce antibodies against the toxoid. Some of these B cells and T cells transform into memory cells, which can rapidly generate a strong immune response upon re-exposure to the same toxoid. When the body encounters the actual toxin, the pre-produced antibodies can quickly neutralize the toxin, preventing it from causing disease. According to the World Health Organization (WHO), by 2015, the number of officially recorded cases of diphtheria had decreased to 4,500, a significant reduction from nearly 100,000 recorded cases in 1980 12. The vaccine is technologically mature and inexpensive, and its safety and efficacy have been proven with long-term use.

#### Conjugate vaccines

Some bacteria, such as *Streptococcus pneumoniae*, have a large number of polysaccharides on their surface that encapsulate them. Polysaccharide vaccines are poorly immunogenic and can only induce an immune response without forming an immune memory for future protection. Exotoxins are chemically treated (e.g., formaldehyde inactivation) to produce toxoids that retain antigenicity but lose toxicity. Toxoids are covalently bonded to polysaccharide antigens or other weakly immunogenic antigens to form a conjugate vaccine. This design enhances the immune response against weakly immunogenic antigens (e.g., bacterial polysaccharides), the toxoid provides a strong T-cell-dependent immune stimulus, and the polysaccharide antigen induces a specific immune response, which induces the production of high-affinity antibodies against the polysaccharide antigen and memory B cells, which improves vaccine efficacy and long-term protection 13. It is clinically important in children and other populations with an incompletely developed immune system.

Representative vaccines include the pneumococcal conjugate vaccine (PCV), which utilizes diphtheria toxoid or tetanus toxoid as a carrier, combined with polysaccharide antigen of *Streptococcus pneumoniae*, used for the prevention of pneumonia, meningitis, and sepsis; Meningococcal Conjugate Vaccine (PCV), which combines *Neisseria meningitidis* polysaccharide antigen with diphtheria toxoid or tetanus toxoid and is widely used to immunize children; *Haemophilus influenzae* type b conjugate vaccine (Hib), which combines the polysaccharide antigen of *Haemophilus influenzae* with tetanus toxoid (TT) for the prevention of pneumonia and meningitis in children.

These vaccines increase immunogenicity so that the polysaccharide antigen activates a T cell-dependent immune response, enhances the memory response, provides long-lasting immunity, is suitable for use in children, and is particularly effective in immunizing children under 5 years of age. However, the disadvantages include complex production processes with possible batch-to-batch variation; preparation requires the use of toxins, increasing production safety requirements; and chemical modification may result in the loss or change of certain antigenic epitopes.

#### Fusion protein vaccines

The antigenic portion of an exotoxin is fused with other functional proteins (e.g., cytokines or carrier proteins) to improve the immune response or specificity against a particular pathogen. For example, fusion protein vaccines are constructed by fusing exotoxins with immune-enhancing molecules (e.g., IL-2) 14. Its advantages include a stronger immune response and its applicability to specific diseases (e.g., tumor vaccines). However, their development is complex and expensive. Taking the fusion protein vaccine of diphtheria toxin as an example, diphtheria toxin consists of two subunits, A and B. The A subunit is toxic, while the B subunit is responsible for binding to cells. Through genetic engineering, the toxic part of the A subunit is removed, retaining the immunogenicity of the B subunit, which is then fused with other antigenic proteins to form a fusion protein vaccine. The B subunit can stimulate the immune system to produce antibodies. By removing the toxic part of the A subunit, toxic reactions are avoided, and the antigenic protein in the fusion protein can elicit a stronger immune response, providing protection against the target pathogen. The fusion protein vaccine offers safe and effective immune protection by retaining immunogenicity, eliminating toxicity, and enhancing the immune response.

#### Genetically Engineered Vaccines (GEVs)

GEVs for exotoxins are a class of vaccines prepared through genetic engineering technology using the coding gene of the exotoxin or its modified sequence. This type of vaccine retains the advantage of the strong antigenicity of exotoxin, while reducing its toxicity through genetic modification to safely activate the body's immune response. GEVs are safer and more efficient than the traditional toxoid vaccines. The current preparation of GEVs can be prepared in two ways. The first is genetic modification, which uses genetic engineering techniques to eliminate toxic activity (e.g., destroying the active site) by targeted mutation or truncation of genes encoding the exotoxin. For example, by altering the EF-2 binding site of DT, antigenicity was retained but catalytic activity was lost. The second is expression by recombinant technology: the modified toxin gene is inserted into an expression vector to efficiently express non-toxic exotoxin proteins in bacterial, yeast, or mammalian cells. Recombinant toxin proteins can be used alone or in combination with adjuvants to enhance immunization. For example, the acellular pertussis vaccine is produced by genetically engineering a non-toxic form of the pertussis toxin, which serves as a component of the acellular vaccine to prevent pertussis^15^.

GEVs have a negligible risk of toxicity, higher safety, and high production efficiency and can be standardized. However, the technical requirements are high, and the production cost is higher than that of the traditional toxoid vaccines.

#### mRNA vaccine

mRNA vaccine technology has made remarkable progress in recent years, not only in the prevention of viral diseases has shown great potential but also began to be applied to the prevention and control of bacterial infections, especially the research of mRNA vaccines against bacterial exotoxins. Such vaccines combine the advantages of mRNA vaccines with the efficient immunogenicity of exotoxin antigens and have become the frontier of vaccine research. A novel vaccine technology that induces an immune response by delivering mRNA encoding an exotoxin antigen to the host cell and utilizing the host cell's translation mechanism to express the antigen. Design of mRNAs encoding exotoxin antigens or modified versions thereof (e.g., non-toxic mutant toxins or antigenic epitopes) with optimized sequences (e.g., incorporation of a 5' cap structure, modified UTR sequences, and polyadenylate tails) to improve translation efficiency and stability. Lipid nanoparticles (LNPs) are used to encapsulate mRNA to protect it from degradation and promote cellular uptake; upon delivery, the mRNA is translated into an exotoxin antigen in the host cytoplasm 16. The translated exotoxin antigen is secreted or displayed on the cell surface, activating host T and B cells, triggering a strong cellular and humoral immune response, and the production of neutralizing antibodies against the exotoxin.

The exotoxins secreted by *Pseudomonas aeruginosa* are highly toxic to the host. Researchers have developed mRNA vaccines encoding PcrV and OprF-I proteins and tested them in a mouse model. The results showed that these mRNA vaccines could induce a strong immune response and reduce bacterial load and inflammatory response, thus effectively protecting mice from infection 17.

The vaccine was developed quickly and flexibly, and the toxin production process was simplified by eliminating the need for the direct handling of live toxins. However, the instability of mRNA and cold chain transportation must be overcome.

#### DNA vaccines

DNA vaccines are manufactured using DNA technology, in which the DNA encodes the antigenic portion of a bacterial exotoxin. This DNA is delivered to the body's cells through a delivery system, and the cells transcribe and translate it into an antigen that triggers the immune system to produce a specific immune response. Genes encoding exotoxin antigens, such as *pseudomonas exotoxin* A and PcrV, are genetically engineered to be inserted into plasmid DNA, which can carry either a complete or modified version of the exotoxin antigen, usually the nontoxic or attenuated portion of the toxin, in order to elicit an immune response without triggering toxicity 18. Delivery is usually achieved by intramuscular injection or electroporation (an electric shock that temporarily opens the cell membrane to allow DNA to enter the cell), or by using carriers such as nanoparticles and liposomes to help DNA enter the cell 19. After DNA enters the cell, it is transcribed in the nucleus or cytoplasm of the cell as mRNA, which is followed by translation of the exotoxin antigen. Exotoxin antigens activate T and B cells, inducing cellular and humoral immunities. The immune response involves the production of neutralizing antibodies against exotoxins and can activate CD8^+^ T cells that recognize and kill infected cells expressing the antigen.

DNA vaccines mimic natural infection through endogenous antigen expression and can stimulate a strong immune response; they are safe and do not involve the use of toxins or viruses, avoiding possible safety issues with traditional exotoxin vaccines, and the selection of antigens can be precisely controlled through genetic engineering to reduce the risk of adverse reactions. They are simple and quick to produce, have a shorter production cycle for DNA vaccines, and do not require large-scale pathogen DNA vaccines to induce long-lasting immune memory and provide long-term immune protection through continuous expression of antigens in the body. They are widely applicable to a variety of bacterial toxins, such as diphtheria toxin, tetanus toxin, and cholera toxin, and can be used in combination with other vaccines to form a multifunctional immune response. However, the delivery efficiency of DNA vaccines remains a challenge, especially when using non-invasive delivery methods, where the efficiency of DNA entry into cells may be low, requiring the use of techniques such as electroporation to enhance DNA uptake. However, these methods may introduce additional operational complexity or discomfort. Despite the ability of DNA vaccines to elicit an immune response, the immune response may be insufficient, especially in the absence of T-cell stimulation, and optimization of vaccine design or combination of adjuvants is needed to enhance the immune response. Although DNA vaccines are more stable than protein vaccines, some vaccines still need to be stored at low temperatures to maintain their stability and efficacy. Long-term safety and efficacy studies are lacking** (Table 1)**.

### Targeted therapy applications

**Figure 2:**
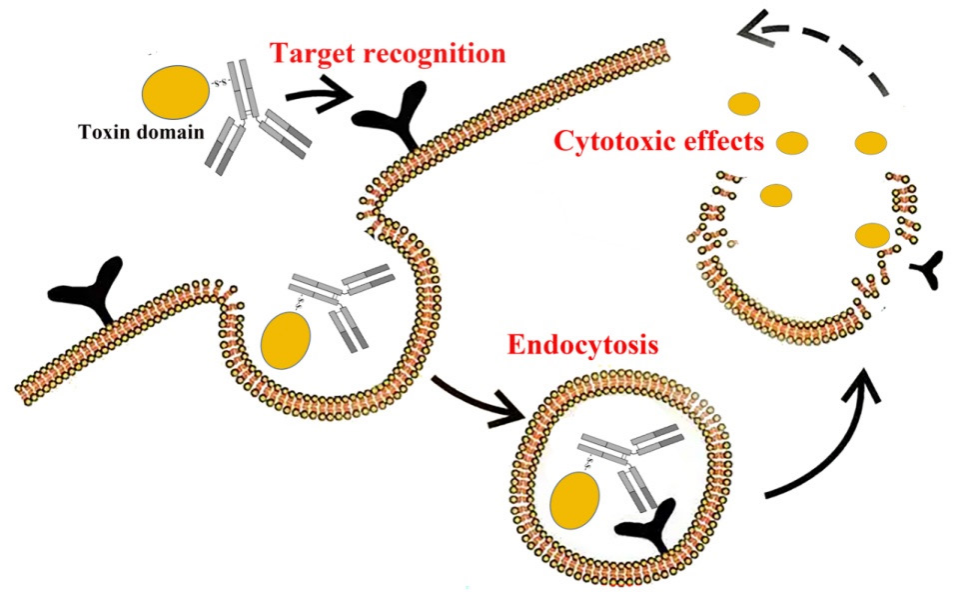
How immunotoxins work.

Immunotoxins (ITs) are a class of fusion proteins that destroy target cells by binding to specific antigens or receptors and delivering cytotoxicity and are widely used in areas such as cancer therapy 20. It consists of a targeting fragment and toxicity fragment. The targeting fragments are usually antibodies, antibody fragments, or ligands of the immune system that can bind cancer cell-specific antigens or receptors. The toxic fragment is usually the toxic portion of a bacterial exotoxin (e.g., diphtheria toxin, tetanus toxin, or certain enzymes). ITs usually work in the following steps: Target Recognition: The ITs recognize and bind specific antigens on the surface of the target cell through its antibody component. Endocytosis: After antigen-antibody binding, ITs enter the cell through receptor-mediated endocytosis. Cytotoxicity: After entering the cell, the toxin portion of ITs exerts a toxic effect, usually killing the target cell by inhibiting protein synthesis or destroying the cell structure** (Fig. 2)**.

Bacterial exotoxins show unique potential for targeted tumor therapy. DT has a potent cell-killing ability and is often used as a prototype for targeting the killing of cancerous or virus-infected cells. ITs are hybrid fusion proteins containing a cell-killing component and a cell-targeting component (antibody or receptor ligand). For example, the fusion of the A fragment of DT to IL-2 recognizes, enters, and kills lymphoma and leukemia cells expressing the IL-2 receptor 21.* Pseudomonas aeruginosa* exotoxin A is a cytotoxin often used in the preparation of ITs, and its mechanism of action is through the inhibition of cellular protein synthesis, thereby inducing apoptosis in target cells. ITs constructed using *Pseudomonas aeruginosa* exotoxin A were genetically engineered to fuse antibody fragments capable of specifically recognizing antigens on the surface of tumor cells with the exotoxin, enabling the ITs to precisely target tumor cells 22,23. A light-controlled gene expression *Pseudomonas aeruginosa* exotoxin-targeted delivery system has been developed for the treatment of breast cancer lung metastasis 24. This system uses nanoparticles to precisely deliver plasmids carrying exotoxin genes to tumor cells, tumor-associated fibroblasts, and tumor neovascular cells, and spatiotemporally and spatially controllable expression of the exotoxin under the control of blue-light irradiation to regulate the tumor microenvironment, which not only inhibits the growth and metastasis of tumor cells but also reduces the toxic side effects on normal tissues. In a therapeutic study of hepatocellular carcinoma, the construction of ITs carrying *Pseudomonas aeruginosa* exotoxin PE38 gene,* Streptococcus* albumin-binding domain (ABD), and targeting human nanobody (HN3), which is able to act specifically on hepatocellular carcinoma cells, is a highly effective therapeutic drug for hepatocellular carcinoma 25.

Fusion with monoclonal antibodies (e.g., antibodies against tumor-associated antigens, such as HER2 and CD22) or other targeting ligands (e.g., IL-2) creates ITs capable of specifically targeting specific tumor cells. It has shown promising results in clinical studies of a variety of cancers and immune diseases, including leukemia, lymphoma, melanoma, and mesothelioma. ITs that have been approved by the U.S. Food and Drug Administration (FDA) for use in humans include denileukin diftitox (FDA Approval: 1999), tagraxofusp (FDA Approval: 2018), and moxetumomab pasudotox (FDA Approval: 2018), the first two of which are DT in a truncated form, and the third is a truncated form of *Pseudomonas aeruginosa* exotoxin A 26,27. These IT therapeutics have revolutionized the treatment options for prostate, breast, bladder, ovarian, colorectal, and pancreatic cancers, as well as leukemia and melanoma.

### Exotoxins as a basis for drug development

Certain bacterial exotoxins can become the basis for the development of new drugs after modification, taking the application of botulinum toxin in the field of medical cosmetology as an example. Botulinum toxin is a powerful neurotoxin produced by *Clostridium botulinum*, and its original effect is to block the release of acetylcholine at the neuromuscular junction, resulting in muscle relaxation and paralysis. It was initially used in the field of medicine for the treatment of muscle spasms and other diseases. However, in medical aesthetics, researchers have utilized its muscle relaxation properties to modify and precisely control botulinum toxin to develop products for wrinkle reduction, face slimming, and other aesthetic programs 28. In wrinkle reduction applications, when botulinum toxin is injected into specific muscles of the face (e.g., muscles in the areas of frown lines, crow's feet), it specifically binds to and inhibits the release of acetylcholine from the nerve endings, thus relaxing the over-contracted muscles and reducing the formation of wrinkles. In terms of the mechanism of action, the light chain of botulinum toxin can enter nerve cells and cleave a protein called SNAP-25, which is essential for the release of acetylcholine. Its cleavage results in improper release of acetylcholine, which in turn prevents the muscle from receiving contraction signals and achieves muscle relaxation.

The combination of bacterial exotoxins and nanomaterials has great potential in the field of nanotechnology. Targeted delivery and controlled release of exotoxins can be achieved by precisely modifying or wrapping exotoxins onto nanocarriers. For example, in tumor therapy, intelligent drug delivery systems constructed using nanotechnology can enable exotoxins to act more precisely on tumor cells, reduce damage to normal tissues, and improve therapeutic effects. The unique properties of nanomaterials, such as high specific surface area and good biocompatibility, can also enhance the stability and bioavailability of exotoxins, providing a new way to develop highly effective and low-toxicity tumor therapeutic drugs 29. The development of gene editing technology has also opened new directions for the application of bacterial exotoxins. For example, the CRISPR-Cas system has made major breakthroughs in the field of gene editing, and bacterial exotoxins are expected to be combined with it to realize more accurate and efficient gene editing. For example, certain bacterial exotoxins can act on specific targets within the cell, and by synergizing with the CRISPR-Cas system, target genes can be cut, modified, or regulated more precisely, providing new strategies for the treatment of infectious diseases and cancers 30. In addition, by utilizing the toxic effects of exotoxins on bacteria, novel antibacterial strategies can be developed to modify bacteria using gene editing technology to produce exotoxins with specific functions for use against drug-resistant bacterial infections and to address the growing problem of bacterial drug resistance 31.

## Challenges and coping strategies in the application of bacterial exotoxins

A toxicity risk assessment is crucial for the application of bacterial exotoxins. Owing to the potential toxic effects of exotoxins on human cells, their risks cannot be ignored. For example, botulinum toxin can specifically act on the neuromuscular junction and cause muscle paralysis by blocking the release of acetylcholine. In the field of medical aesthetics, although botulinum toxin is widely used in treatments such as wrinkle removal and face slimming, improper use, such as excessive dosage or inaccurate injection site, may trigger serious adverse reactions, including muscle weakness, dysphagia, and dyspnea, or even life-threatening 32.

The exotoxin portion of immunotoxins used in tumor therapy may be toxic to normal tissue cells, leading to side effects, such as hepatic and renal impairment and immunosuppression 33. Therefore, an accurate assessment of the toxicity risk of exotoxins is a critical step in ensuring their safe application. Currently, the commonly used toxicity risk assessment methods include animal and cellular experiments. In animal experiments, by injecting different doses of exotoxin into experimental animals and observing their physiological responses, pathological changes, mortality, and other indicators, the lethal dose half (LD50) and effective dose half (ED50) of exotoxin were determined to assess toxicity intensity and therapeutic window. Cellular experiments, on the other hand, utilize cell lines cultured in vitro to observe the effects of exotoxin on cell growth, proliferation, apoptosis, metabolism, etc., and to determine the mechanism of its toxic effects on cells and the dose-effect relationship. In addition, modern biotechnological methods, such as gene microarrays and proteomics, can be combined to study the effects of exotoxins on intracellular gene expression and protein synthesis in depth, to reveal the mechanism of their toxic effects at the molecular level, and to provide a basis for more accurate toxicity risk assessment 34.

In addition to the direct toxic effects mentioned above, exotoxins may trigger allergic reactions. In some individuals, the immune system recognizes exotoxins as foreign antigens, resulting in the production of specific antibodies. When re-exposed to exotoxin, the antigen-antibody combination triggers an allergic reaction, which manifests itself in symptoms such as rash, itching, respiratory distress, and anaphylactic shock, the severity of which varies from person to person 35. The mechanism by which allergic reactions occur involves overactivation of the immune system, including the release of biologically active mediators such as histamine, by degranulation of mast cells and basophils, leading to physiological responses such as vasodilation, increased permeability, and smooth muscle contraction. Skin tests, serum-specific IgE assays, and other methods are commonly used to assess the risk of developing allergic reactions. Skin tests involve intradermal injection of a small amount of exotoxin or scratch inoculation on the skin surface of the subject, observing local skin reactions such as redness, swelling, and itching, and determining the possibility of an allergic reaction. Serum-specific IgE testing, however, indirectly assesses an individual's tendency to be allergic to exotoxin by detecting the level of specific IgE antibodies against exotoxin in the blood. For people with a known history of allergy or hypersensitivity, strict allergy screening must be conducted before using exotoxin-related products or undergoing exotoxin treatment. Corresponding preventive measures, such as the use of anti-allergic drugs and desensitization treatment, must be taken to reduce the risk of allergic reactions and ensure patient safety.

A series of safety control measures and standards has been developed and implemented to address the toxicity risks of exotoxins. In terms of dosage control, precise calculation and strict control of the dosage of exotoxin used is key, depending on the type of exotoxin, the field of application, and the target of treatment. In the pharmaceutical industry, strict adherence to good manufacturing practices (GMP) is an important safeguard to ensure the safety and efficacy of exotoxin-related drugs.

## Conclusion and outlook

Bacterial exotoxins have diverse and important applications in the medical field. As a marker for disease diagnosis, early and precise diagnosis of many diseases can be realized by means of immunoassay and fluorescence technology and provide a key basis for timely treatment. In terms of treatment, whether it is the use of antitoxins to neutralize the toxicity of exotoxins in immunotherapy or the use of exotoxin-constructed immunotoxins to accurately fight against tumor cells in tumor-targeted therapy, all of them have opened up a new way of overcoming difficult diseases and have significantly improved the survival rate and quality of life of patients. With the rapid development of science and technology, bacterial exotoxins have shown broad prospects for future research and application, providing innovative ideas and methods for solving many difficult problems.

## Author contributions

Conceptualization: FR and YT; Project Administration: ZY; Supervision: YT; Writing-Original Draft Preparation: YX: Writing-Review & Editing: YT. All authors have read the submitted version of the paper and have approved its submission.

## Figures and Tables

**Table 1:** Types of bacterial exotoxin vaccines and their advantages and disadvantages

Type of vaccine	Principle	Advantages	Disadvantages	Representative vaccines
Toxoid vaccines	De-toxify the toxin produced by the pathogen, retain antigenicity, and produce a specific immune response	High safety, few side effects, specific immune response, suitable for protection against toxin-induced diseases	Only protects against specific toxin-induced diseases; requires regular immunization	Tetanus vaccine, diphtheria vaccine
Conjugate vaccines	Combines polysaccharide antigen of the pathogen with toxoid to enhance the immune response	Enhances the immune response, especially in newborns and young children, and can fight multiple pathogens	May not be as effective in some populations with weakened immune systems; requires precise technology to create antigenic conjugates	Hib vaccine (hemophilic influenza), quadruple vaccine (diphtheria, tetanus, pertussis, polio)
Genetically engineered vaccines	A class of vaccines prepared by genetic engineering techniques utilizing genes coding for exotoxins or their modified sequences	Faster and safer production, eliminates the need for intact pathogens, and adapts to novel pathogens	Co-adjuvants may be required, and production facilities and technology are more demanding	Hepatitis B vaccine (recombinant protein), HPV vaccine (human papillomavirus)
Fusion Protein Vaccines	Fuses antigens of pathogens with proteins that enhance immune response (e.g., immune-enhancing factors) to form new fusion proteins that activate the immune system	May enhance the immune response, more so than vaccines against antigens alone, sometimes enhancing antibody production and T-cell immunity	May trigger a strong immune response, with a complex production process	Influenza vaccines (some types), Sars-cov-2 vaccines (some types of fusion proteins)
mRNA vaccines	By delivering mRNA encoding a pathogen antigen, cells in the body are prompted to express that antigen and activate an immune response	Extremely fast to produce, easy to customize, can respond quickly to emerging pathogens; strong immune effect, widely adaptable	It requires cold-chain storage and transportation, long-term effects still need to be evaluated, and there are large individual differences in immune response	Sars-cov-2 Vaccines (Pfizer/BioNTech, Moderna)
DNA vaccines	The DNA encoding the pathogen antigen is injected directly into the body, and the host cells will synthesize the pathogen antigen and stimulate an immune response	Easy and fast to produce, better immunogenicity, can be rapidly developed against novel pathogens	Delivery is technically difficult and may require co-adjuvants to enhance immunity	Sars-cov-2 Vaccines (COVAXIN, ZyCoV-D)
